# Comparison of negative pressure wound therapy (NPWT) &conventional wound dressings in the open fracture wounds

**DOI:** 10.12669/pjms.321.8568

**Published:** 2016

**Authors:** Hamidreza Arti, Mohsen Khorami, Vahid Ebrahimi-Nejad

**Affiliations:** 1 Prof. Hamidreza Arti, Department of Orthopedic Surgery & Trauma Research Center, School of Medicine, Ahvaz Jundishapur University of Medical Sciences, Ahvaz, Iran; 2 Mohsen Khorami, Assistant Professor, Department of Orthopedic Surgery & Trauma Research Center, School of Medicine, Ahvaz Jundishapur University of Medical Sciences, Ahvaz, Iran; 3 Vahid Ebrahimi-Nejad, Resident, Department of Orthopedic Surgery & Trauma Research Center, School of Medicine, Ahvaz Jundishapur University of Medical Sciences, Ahvaz, Iran

**Keywords:** Wound healing, Negative Pressure Wound Therapy, Conventional Wound Dressings, Infection

## Abstract

**Objective:**

Successful closure is a primary step of treatment in open fracture wounds. Delayed healing or complications can lead to increased treatment duration, costs and disability rates. The aim of this study was to compare Negative Pressure Wound Therapy (NPWT) and conventional wound dressings in patients with open fracture wounds.

**Methods:**

In a prospective randomized clinical trial study, 90 patients with open fractures that were referred for treatment were enrolled between February 2013 to March 2015. Patients were divided into two groups. Group I underwent NPWT and group II underwent conventional wound dressing. Then patients were followed up for one month. Within the one month, the number of dressing change varied based on the extent of the wound. Duration of wound healing, presence of infection and the number of hospitalization days in these patients were recorded and compared at the end of the study between the two groups. Questionnaires and check lists were used to collect data. Analysis was done with SPSS 20_,_ paired sample T-test, and chi-square tests. P<0.05 was considered significant.

**Results:**

There was a significant difference between the rate of wound healing in the group one or NPWT group and group II (conventional wound dressings) P<0.05. There was no significant difference between two groups in incidence of infection (P=0.6).

**Conclusion:**

Using NPWT expedites the healing process of extremity wounds. It is more economical and can be considered as a substitute for the treatment of extremity wounds.

## INTRODUCTION

When the wound meets the fracture hematoma, it is considered an open fracture. Open fracture often results from high energy trauma. Wound management and prevention of infection is a basic step in treatment of open fractures. Early and suitable treatment of open fractures is extremely important as open fractures are associated with increased risk of infection and complications during treatment.^1^,^2^

For assessment and treatment of open fractures in extremities, various factors should be considered such as patient’s condition, fracture type or open fracture classification, antibiotic treatment, wound debridement, fracture fixation, size and site of the wound, the extent of muscle injury. Open fractures are classified according to Gustilo-Anderson classification system.^3^

The infection of open fractures are generally known to be between 0-2% in type I, 2-10% in type II and 10-50% in type III. Antibiotic treatment duration and wound remodeling time are not directly responsible for infection.^4^,^5^ NPWT is a therapy that uses gauze or foam to promote wound healing.^6^ The brand name VAC is the most commonly used NPWT.^7^,^8^ This new technology was introduced by Argenta and Morykwas almost 20 years ago for medical treatment. This method employs negative pressure via a special closed wound dressing by intermittent or continuous application to the wound.^6^ This negative pressure launches a biological effect that subsequently leads to improved healing.^7^ Complex wound treatment is difficult.^9^ Initially, NPWT was identified as an adjunctive therapy to help heal difficult open wounds and many clinical trials have cited the success of this method in open wound treatment. NPWT is an affordable and effective method to help heal wounds.^9^,^10^ The idea NPWT or Vacuum-Assisted Closure (VAC) is currently used as the standard treatment in some institutes. The current literature suggests primary mechanisms of action of the VAC device may include the following:

Drawing the wound edges together,^4^,^5^ stabilization of the wound environment; accelerates wound healing by promoting the formation of granulation tissue, in order to completely close or improve the health of a wound in preparation for a skin graft.^11^ Decrease in wound edema and removal of wound exudate; and microdeformations of the wound surface. Secondary effects include increased angiogenesis, granulation tissue formation, and, in some cases, a decrease in bacterial bioburden. After 3 to 4 days of therapy, bacterial counts in the wound drop.^5^,^12^,^13^ Despite the increasing application of new therapeutic modalities in improving the treatment of wounds such as various dressings, local growth factors, hyperbaric oxygen and local and systemic antiseptic agents, treatment of serious wounds still remain as a clinical enigma.

It seems that employing dressings with suction or negative pressure has a positive effect on treating open fractures and reducing complications.^4^,^11^,^14^ The aim of this study was to evaluate the effect of NPWT dressing on open fracture wounds in comparison with conventional wound dressing.

## METHODS

The study protocol was approved by the ethical committee of Ahvaz Jundishapur University of Medical Sciences and registered in IRCT. All patients signed an informed consent form, before taking part in this study. It is a prospective randomized clinical trial study by simple convenience sampling conducted between February 2013 to March 2015, Of 136 patients with open fracture that were referred to Golestan university hospital, in Ahvaz, Iran for treatment of their open fractures 46 of them were excluded from study hence 90 remaining patients were enrolled. All the patients were divided into two groups. Group I underwent NPWT and group II underwent conventional wound dressing twice a day by senior resident. Vacuum-Assisted Closure Device (VAC) was a machine that produced negative pressure. After detailed debridement of open fractures and obtaining a clean wound with skin and soft tissue loss, sponge foam was placed on the wound. Then the wound was covered by an adhesive drape. Finally, the innerend of a suction tube was inserted in the dead wound space and the outer end of it was connected to the device. Wound dressings were changed usually every 48 hours and negative pressure continued for 10-14 days. pressure was maintained at -125 mm Hg continuously or intermittently 5 minutes on two minutes off. It’s necessary to say that in control group patients were treated with conventional dressings.

Inclusion criteria were persons between the age of 15-55, presence of an open fracture wound type IIIB based on Gustilo-Anderson classification, and accessible clean wound after a meticulous debridement. Exclusion criteria were type I, II or IIIA and IIIC based on Gustilo-Anderson classification, need of vascular repair or reconstruction, presence of multiple fractures in extremities, malnutrition, systemic disease, dermatological disease like psoriasis, immunosuppressive drug consumption, existence of old fracture or implant in the fractured extremity and previous osteomyelitis. Then patients were matched for age, sex and type of open fracture and were assigned to either one of two groups based on random table numbers. Patients were followed up for one month. Patients were advised to come to the hospital for routine checkups after being discharged, and all participants were followed up throughout the study.

Wounds were examined weekly and following, measurements were recorded presence of granulation tissue, wound bed becomes redder, decrease in wound drainage, and decrease in dimensions of wound. V. A.C. Therapy was terminated when adequate granulation base was achieved allowing for change to conventional dressing, split-thickness skin graft, or flap closure.

Within one month, the number of dressing change varied based on the extent of the wound. Wound healing duration, presence of infection and the number of hospitalization days in these patients were recorded and compared at the end of the study between the two groups. Wound infection in this study was defined as purulent discharge from the wound site or positive culture of the wound.^14^,^15^ Data (demographic data, infection, hospitalization day, wound surface reduction) were collected by using a structured questionnaire and analyzed with SPSS_20_ by means of independent t-test for hospital stay, paired samples t-test for wound surface reduction and chi-square test for infection differences between two groups. P<0.05 was considered significant.

## RESULTS

In this clinical trial study, 90 patients were enrolled and were divided into two treatment groups. These included 22 (22.4%) females and 68 (75.6%) males who were randomly and evenly assigned to two treatment groups (Table-I).

**Table-I:** Distribution kind of bone Fractures based on treatment methods.

Treatment method	Tibia and Fibula	Femur	Humerus	Radius & Ulna	Total
Group I g(NWPT)	30	10	2	3	45
Group II (Conventional Dressing)	30	10	3	2	45

The mean age of participants was 31.86 ± 9.7 years the minimum age among the patients was 16 and the maximum age was 53 years. Age distribution in both groups was almost similar and was not statistically significant difference (P=0.7). There was no significant difference between two groups in terms of demographic data (age, sex, weight, height) (P=0.071) Classification type was assessed for all participant patients by using Gustilo-Anderson classification system. Pattern of fractures type and fixation method, location and size of wound in two groups were similar.

At the end of the study, the patients in the two groups were assessed for the infection rate. In the group I or NPWT group, 3 cases of infection were found that a patient had deep infection and in the group II or conventional dressing group 4 cases of infection seen that a patient had deep infection. The difference of infection between the groups I and II were not statistically significant by chi-square test (p=0.6).

Mean duration of hospital stay for wound preparation for coverage by skin graft or flap was 10.5±2.8 days for all enrolled patients which was 9.7±2.3 and 11.2±3.1 for group I or NPWT group and group II (conventional dressing group) respectively. The difference between the groups I and II regarding reduction in hospital stay were statistically significant. On the other hand T-test showed hospital stay in NPWT group was less than conventional dressing group (P=0.01). Wound surface reduction were 19% in group I and 6% in group II. The results of paired sample t-test showed a significant difference in wound surface before and after treatment in group I (P=0.001). Also, the results of paired sample t-test did not show a significant difference in wound surface before and after treatment in group II(P=0.76). Independent samples t-test showed a significant difference between group I and II in wound surface reduction (P=0.011). Fifteen patients in group II and 5 patients in group I needed skin graft and one patient in each groups that their wound location were in distal leg needed flap coverage.

## DISCUSSION

Negative Pressure Wound Therapy (NPWT) is a method for dressing management in soft tissues of high-grade fractures.^5^ This method enhances protein and collagen production and diminishes bacteria colonization. Consequently it is beneficial in treating acute and chronic wounds.

In our study, one group of patients with open fracture was managed by NPWT dressing and the other group by conventional dressing. The duration of wound healing, the number of hospitalization days and incidence of infection was registered for both groups, and then compared. There was no significant difference between two groups in incidence of infection. Mean duration of hospital stay for NPWT group was less than group II. There was significant difference in wound surface before and after treatment in NPWT group and we have seen significant difference between group I and II in wound surface reduction. Open fractures are susceptible to infection; however infection rarely occurs in closed fractures as well.^15^-^17^ Commonly, wound infection permeates into the bone which results in osteomyelitis and is a critical factor in hindering or delaying wound closure. The rate of infection development in open fractures depends on various forms of fracture, type of intervention, antibiotic therapy and the patient’s condition.^18^,^19^-^21^ In type IIIB and IIIC of open fracture bone stabilization is performed by external fixation and.^20^-^22^ In our study, the group with conventional dressing presented with 4 cases of infection and in the NPWT dressing group 3 infection cases were found, all of which belonged to tibia and fibula fractures. The latter group however presented with fewer cases of infection but did not show any significant difference with the former. It seems that the development of infection relies on numerous factors and dressing type is not the sole determinant. Dedmond et al. showed that the infection rate was 12.5% for grade/type IIIA open fractures, 45.8% for grade/type IIIB, and 50% for grade/type IIIC. They also concluded that infection and nonunion rates with the use of NPWT for temporary coverage of wounds associated with grade/type III open tibial shaft fractures are similar to those of historical controls, but this technique may be beneficial in decreasing the need for free tissue transfer or rotational muscle flap coverage.^23^ Blum et al. found that use of NPWT could reduce the risk of deep infection by almost 80%.^22^ In our study, open fractures of type I and II Gustilo were omitted which have lower risk of infection. A study on factors affecting infection development in patients with open fractures was conducted. In this study the researcher compared patients who suffered infection with the control group who were infection-free. They evaluated and compared the two groups in terms of risk factors for developing infection. Smoking, mean smoking time, mean number of transfusions, the time of debridement and diabetes in the two groups had a significant difference but factors such as body mass index(BMI), sex, and age had no influence in the rate of infection occurrence.^15^ However, some studies indicate age as an important factor in wound healing and believe that with age increase, sensorineural function which has a major role in tissue reconstruction, declines.^24^,^25^ The mean hospital stay for patients treated with Negative Pressure Wound Therapy (NPWT) dressing was less than those of conventional dressing. Regarding enhancement in wound healing with NPWT method, the result of several studies corroborate our results. For instance, in a study on open fracture wounds of femur bone achieved similar results with our study. A systematic review article also concluded that NPWT results in reduced wound size and formation of granulation tissue that is similar to our study.^25^ Furthermore, Argenta and Morykwas and others who published their study results on would restoration under negative pressure, believed that negative pressure of 125 mm Hg leads to increased vascularity and blood flow 4 times as usual in the wound bed and thus accelerating wound healing.^14^,^26^ The difference of our study with those mentioned above was in using suction device or vacuum. In our study, sponge was employed and it was different from previous works only in terms of reducing infection risk and the effect it has on enhancing recovery from infection. Overall, it seems that this method can be used as a simple and accessible one with the aim of shortening wound healing time and reducing complications in open fracture wounds’ dressings. Further studies can reveal characteristics of this therapeutic modality in finer details.

## CONCLUSION

We had similar results like other studies on NPWT method on expediting the rate of wound healing. Given the simplicity and low cost of this method and lack of distinctive side effects, it could be considered as an appropriate dressing. Further, by reducing hospitalization days, this method could reduce the costs imposed on both the patient and the health system. Also, this method can lead to better outcome in wound surface before and after treatment in NPWT group and significant change in wound surface reduction. We suggest a similar study with Negative Pressure Wound Therapy (NPWT) dressing to be conducted on more patients with infection following an open fracture and in that study the time of infection recovery and wound healing should be closely compared with conventional dressing.
